# Interfacial Redox Recycling Nanocatalysts with Ultrahigh Peroxidase Activity for Colorimetric Sensing Applications

**DOI:** 10.1021/acsanm.6c01249

**Published:** 2026-05-29

**Authors:** Santimukul Santra, Eniola Arogunyo, Rahab Kanogo, Abigail Teitelbaum, Caroline Gichuru, Fei Wang, Rishi Patel, Tuhina Banerjee

**Affiliations:** 1 Department of Chemistry and Biochemistry, 7471 Missouri State University, 901 S. National Avenue, Springfield, Missouri 65897, United States; 2 Department of Chemistry, 6594 Pittsburg State University, 1701 S. Broadway Street, Pittsburg, Kansas 66762, United States; 3 Jordan Valley Innovation Center, 7471 Missouri State University, 542 N. Boonville Avenue, Springfield, Missouri 65806, United States

**Keywords:** nanozyme, redox recycling, plasmonic nanoceria, density functional theory, *E. coli* O157:H7

## Abstract

For the past few years, conventional peroxidase mimics of nanoscale materials have found limited applications because of their low catalytic activity. Hence, it is desirable to control and tune their physicochemical properties through precise engineering to achieve a superior catalytic efficiency. Herein, we demonstrate an efficient strategy for substantially improving the peroxidase-mimetic activity of nanomaterials, particularly those that exhibit mixed redox states. One of these synthesized redox-active nanostructures is plasmonic nanoceria (PNC), which consists of a cerium oxide core with several encapsulated plasmonic gold nanoparticles within its poly­(acrylic acid) polymer coating. PNC nanostructures exhibit enhanced catalytic activity with a *K*_cat_ value of 10^6^ s^–1^, 10^3^-fold higher than that of natural enzymes. Importantly, the catalytic activity of PNC was present over a wide range of temperatures and pH. Density functional theory (DFT) calculations revealed that efficient electron transfer from gold (Au) to cerium (Ce) atoms in the PNC significantly boosts its catalytic activity. Using *Escherichia coli* O157:H7 as a target pathogen, it is demonstrated that when PNC is applied as a peroxidase mimic for an enzyme-linked immunosorbent assay (ELISA), lower limits of detection are achieved than in conventional assays employing natural enzymes.

## Graphical abstract

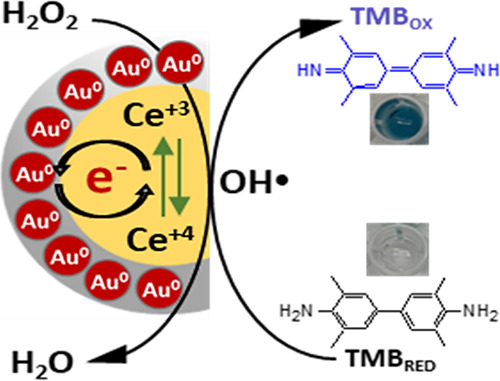

## Introduction

For the past few years, nanoscale materials exhibiting enzyme-like properties, often referred to as “nanozymes”, have been used extensively as an alternative to natural enzymes.^1–10^ Since 2007, when peroxidase-like properties of Fe_3_O_4_ were demonstrated,^5^ over 300 types of nanomaterials^11^ with tailorable morphologies and intrinsic enzymatic properties have been discovered. Owing to enhanced stability, lower production costs, and multifunctionality than natural enzymes, nanozymes have been employed for widespread applications ranging from biosensing to environmental protection and, in recent years, for precise cancer detection and therapy. Despite enormous developments in the field of nanozymes, poor specificity, stability, and limited catalytic efficiency still constitute major challenges.^12,13^ Particularly, metal-based nanozymes with mixed valence states, which are synthesized using a high-temperature solvo-thermal process, display insufficient catalytic efficiency. Critical parameters of the catalytic activity of such nanozymes strongly rely on the experimental conditions and efficiency of the redox cycling of their mixed valence states.^14–23^

Among various metal-based artificial enzymes, cerium oxide (CeO_2_) nanozymes have enormous potential to be more biocompatible and less toxic. In addition, their tailorable physicochemical properties, controllable size, morphologies, and ability to exist in dual reversible oxidation states (Ce^3+^/Ce^4+^) endow them with diverse enzyme-like properties such as the activities of catalases, oxidases, superoxide dismutases, and peroxidase mimetics. Some of these enzymatic activities, particularly peroxidase-like catalytic activity, have been shown to proceed via a Fenton-like reaction.^24,25^ The overarching goal through the years has been to enhance the catalytic efficiency of CeO_2_ nanozymes. There have been some attempts to correlate catalytic activity with changes in its structure. Experimental evidence suggests that improved catalytic activity in CeO_2_ NPs is associated with a higher fraction of Ce^3+^ sites and oxygen vacancies^26–32^ that arise when the size of CeO_2_ is reduced to nanoscale. In one of these research studies, it was shown that porous nanorods of ceria exhibited high peroxidase-mimetic activity (*K*_cat_ ∼10^4^ s^–1^),^32^ which resulted from a large surface area and a higher level of oxygen vacancy. To increase surface abundance of structural defects in CeO_2_ nanozymes, several approaches have been introduced. These include synthesis of Co-doped mesoporous CeO_2_, Mn/Zr-co-doped CeO_2_, and Cu-doped CeO_2_.^31^ However, doping of CeO_2_ with a heteroatom did not result in the activation of substrate H_2_O_2_ due to the generation of a metastable oxygen vacancy.^33^ Based on these findings, it was concluded that the electron density of surface Ce species played an important role for the activation of a surface oxygen vacancy for efficient catalytic activity. Although CeO_2_ nanozymes have been extensively studied for the past few years, their catalytic efficiency is ultimately limited (*K*_cat_ ∼10^3^–10^4^ s^–1^) due to their inert redox recycling between Ce^3+^/Ce^4+^ valence states. Thus, the development of facile and efficient methods for accelerating the redox cycles is crucial.

In this work, we introduce a new design based on hybrid PNC nanostructures for substantially improving the catalytic efficiency of CeO_2_-based nanozymes. The working principle and mechanism of higher peroxidase-like activity is shown in Figure 1. Increased synergy between gold nanoparticles (GNPs) and CeO_2_ NPs due to optical-plasmon coupling interactions is expected to mediate the electron transfer process from the Au atom to Ce and thus result in faster recycling of Ce^3+^ from Ce^4+^. Ce^3+^ then acts as a catalytic center and transfers the electron to H_2_O_2_, which dissociates into reactive hydroxyl ions (OH•) that oxidize colorless TMB into a blue-colored product. Moreover, the catalytic activity of the PNC nanostructures is maximized by tuning the number of loaded plasmonic nanoparticles (GNPs) while keeping the morphology of the nanoparticle unchanged. Our experimental results indicate that such nanoscale materials display a much higher peroxidase-like activity (*K*_cat_ ∼10^6^ s^–1^) that results in an intense blue-color signal due to the instantaneous oxidation of chromogenic substrate TMB in the presence of H_2_O_2_. Importantly, density functional theory (DFT) calculations further provide evidence that higher catalytic activity occurs due to accelerated redox cycling between Ce^3+^ and Ce^4+^, induced via electron transfer from the Au to the Ce atom. As proof-of-concept studies, PNC nanostructures were applied to an enzyme-linked immunosorbent assay (ELISA) of *Escherichia coli* O157:H7 (Scheme 1), which achieved lower detection limits, as low as 10 colony forming units (CFUs), compared to conventional peroxidase-based assays.

**Figure 1:**
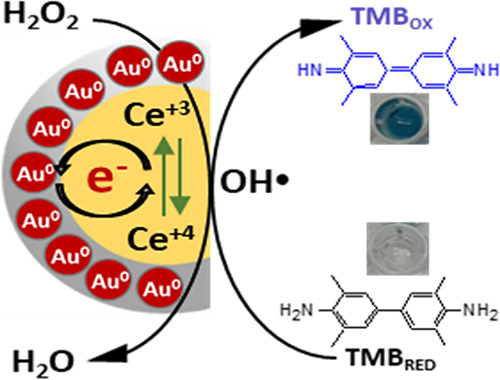
Schematic illustration of the working principle of plasmonic nanoceria (PNC).

**Scheme 1:**
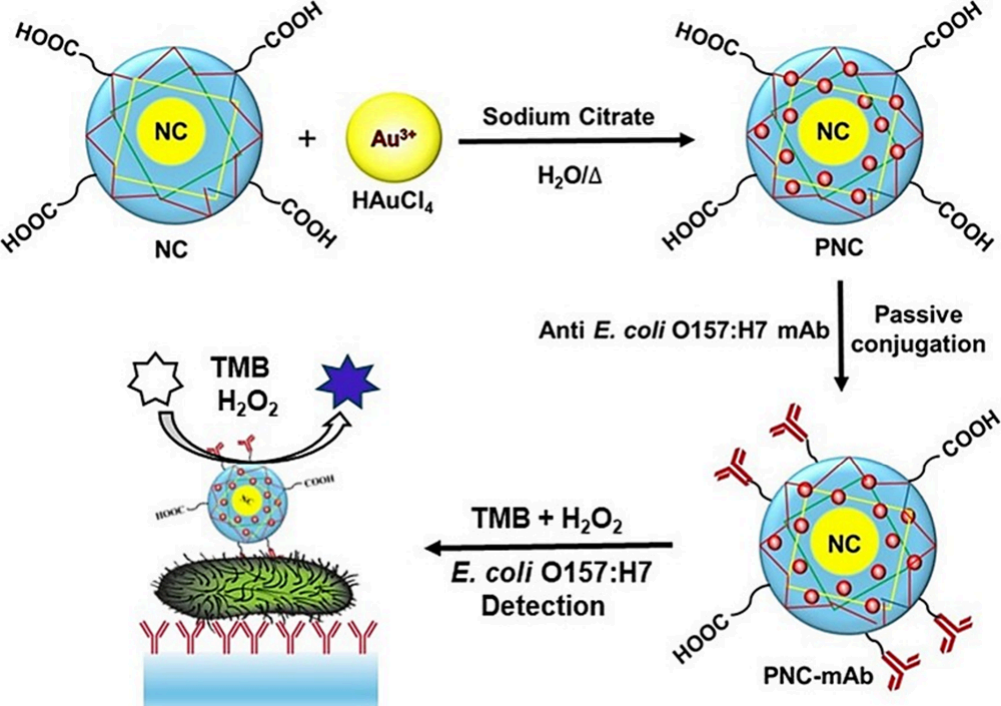
Stepwise PNC Formulation and Anti-*E. coli* O157:H7 mAb Conjugation for Detection of a Food-Borne Pathogen, *E. coli* O157:H7.

## Experimental Section

### Materials

Bovine serum albumin (BSA), phosphate-buffered saline (PBS), ammonium hydroxide (NH_4_OH), cerium oxide, 3,3′,5,5′-tetramethylbenzidine (TMB), hydrogen peroxide 30% (H_2_O_2_), and horseradish peroxidase (HRP) were obtained from Fisher Scientific, and hydrogen tetrachloaurate­(III) trihydrate (HAuCl_4_·3H_2_O) was bought from Strem Chemicals. Polyacrylic acid (PAA) was purchased from Sigma-Aldrich Chemical. The dialysis bag (MWCO 6–8 K) was purchased from Spectrum Laboratory. A pair of monoclonal antibodies mAbs for *E. coli* O157:H7 detection and LFA A/G strips were acquired from Abcam. The LFA materials kit was purchased from DCN diagnostics. Bacterial strain *E. coli* O157:H7 was purchased from the American Type Culture Collection (ATCC). The Lightning-Link HRP antibody labeling kit was obtained from Abcam.

### Instrumentations

The hydrodynamic diameter and zeta potential of nanoparticles were determined using Malvern’s Zetasizer ZS90. The SpectraMax M5 plate reader was used for UV–vis absorbance and fluorescence measurements. Transmission electron microscopy (TEM) images were acquired on JEOL JEM-2100 equipped with the Bruker Quantax 200 energy-dispersive X-ray microanalysis (EDS) system used for elemental mapping of synthesized nanomaterials. XPS measurements were done on a Nexsa XPS surface analysis system from Thermo Fisher Scientific using a microfocused, monochromated, low-power, Al Kα (1486.6 eV) X-ray radiation source. A Rigaku MiniFlex 6G diffractometer with Cu Kα radiation was used for conducting XRD experiments. FT-IR data was obtained using a Vertex 70 Fourier transform infrared/Raman spectrometer from Bruker.

### Preparation of Cerium Oxide Nanoparticles (NC)

Two solutions were prepared initially. Solution 1 containing 0.9 g of cerium nitrate was dissolved in 2.5 mL of DI water. Solution 2 was prepared by adding 0.9 g of PAA in 10 mL of DI water. After preparing both the solutions, 30 mL of ammonium hydroxide was added into a 150 mL conical flask and was stirred at high speed. Next, solution 1 was added to the stirring solution, and immediately solution 2 was added and a color change was observed from colorless to brown. The solution was stirred for 24 h until a deep-yellow solution was observed and was centrifuged at 3000 × *g*. The supernatant was collected and dialyzed (molecular weight cutoff: 6–8 K) in DI water and PBS (pH 7.4) overnight to remove the unreacted mixture. The purified CeO_2_ NPs were stored at room temperature, and the estimated particle concentration was 3.0 × 10^3^ mM.

### Synthesis of PNC Nanostructures

Synthesis of PNC involved two steps. In a standard procedure, CeO_2_ NPs (NC) were synthesized, followed by *in situ* GNP preparation using the Turkevich method. Briefly, 2 mL of the 5 mM HAuCl_4_ was added to a conical flask filled with 10 mL of CeO_2_ NPs (5 mM) and 7.0 mL of deionized water. The solution was heated at 100 °C with continuous stirring and left to boil for 10 min, after which 2 mL of 0.5% sodium citrate was added and continued boiling until a color change from purple to ruby red color was observed. To achieve different amounts of loaded GNPs within the PAA coatings of CeO_2_ NPs, the concentration of HAuCl_4_ was varied.

### Bacterial Culture

Freeze-dried pellets of the *E. coli* O157:H7 strain were obtained from ATCC. Pellets were resuspended with 5 mL of nutrient broth, and 100 μL of resuspended pellet was added onto the agar plate. Spreading was done using a sterile bent glass rod, and plates were incubated for 24 h at 37 °C. On the next day, isolated colonies were picked and 35 mL of nutrient broth was added, followed by an overnight incubation at 37 °C. Bacterial solution was then centrifuged at 6000 × *g* rpm for 10 min. The pellet was washed with 1× PBS (pH 7.4), and the optical density of the solution was adjusted to 0.1. Confirmation of the bacterial concentration in the solution was done by a plate counting method.

### Kinetic Analysis

Peroxidase-mimetic activities of PNC nanostructures were determined using steady-state kinetic assays. All of the assays were performed in 4.5 mL cuvettes at room temperature and with 0.2 M sodium acetate buffer (pH 4.0). Following addition of TMB and H_2_O_2_ to the reaction solution containing nanoparticles, the absorbance of the resulting solution was measured at 652 nm as a function of time at an interval of 3 s for 5 min using the GENESYS 150 spectrophotometer. From these measurements, absorbance versus time plots were constructed, and the slopes derived were used for the calculation of initial velocity. Curves of initial velocity versus TMB concentrations were fitted using Origin Pro 2019 with the Michaelis–Menten enzyme kinetic model and Levenberg–Marquardt iteration graph, and kinetic parameters were determined, including maximum initial velocity (*V*_max_), Michaelis–Menten constant (*K*_m_), and catalytic constant (*K*_cat_).

### DFT Calculations

First-principle calculations were done using the Vienna Ab initio Simulation Package^34–37^ on the High Performance Computing Center, Missouri University of Science and Technology.^38^ The details of the computational model structures, CeO_2_ (111) slabs, and Au on CeO_2_ (111) slabs are introduced in Results and Discussion. For all calculations, we used a 3 × 3 × 1 Monkhorst mesh^39^ to sample the first Brillouin zone. Electronic exchange and correlation were treated with the Perdew–Burke–Ernzerhof (PBE)^40^ generalized gradient approximation. An on-site Hubbard *U* parameter^41^ of 5.0 eV was used for Ce 4f states using the approach introduced by Dudarev et al.^42^ Spin polarization was applied assuming a ferromagnetic ordering. Atomic position optimization was performed with the conjugate gradient algorithm. Bader’s charge analysis was done with the optimized structures.

### PNC Functionalization and Flocculation Experiments

PNC (OD_542_ = 1.0) solutions were adjusted to pH 9.0 with 0.1 M potassium carbonate. Subsequently, 0.1 mL of antibody (concentration 1–20 μg/mL) was added. Following mixing, conjugate solutions were incubated for 10 min at room temperature. Finally, 5% BSA was added to the conjugate mixture to block nonspecific binding, centrifuged at 8000 × *g* for 10 min at 4 °C to remove unbound antibody, and finally suspended in 5% BSA carbonate buffer. To determine the stabilizing concentration of mAb for conjugation, flocculation experiments were performed. To each of these conjugates, 10% NaCl was added and OD_580_ was measured to estimate the optimum concentration of the antibody for PNC conjugate synthesis. Additionally, successful conjugation of the antibody was further verified using the conjugation checking kit (ab 236554 from Abcam).

### Sandwich ELISA

For conducting sandwich ELISA in s, 96-well microplates were coated with 100 μL of pAb-*E. coli* O157:H7 capture antibody (5 μg/mL) in coating buffer overnight at 4 °C followed by blocking. In each well, different CFUs of *E. coli* O157:H7 were added and incubated for 2 h at 37 °C. Unbound CFUs were removed by rinsing the plates with washing buffer three times. The optimized concentration of PNC–mAb conjugates was added to each well and allowed to incubate for 2 h at room temperature. Finally, 800 μM TMB and 210 mM H_2_O_2_ were added to each well and incubated for 10 min and absorbance was measured at 652 nm. For a direct comparison between HRP-based sandwich ELISA and PNC-based ELISA, the same capture/detection antibodies were used, and the assay procedure was kept similar. Differences included the replacement of PNC–mAb conjugates with HRP–mAb conjugates. Conjugation of antibodies with HRP was performed using the Lightning-Link HRP antibody labeling kit. For evaluating the detection sensitivity of PNC-based sandwich ELISA in complex food matrices, milk (2% in 1× PBS) was spiked with *E. coli* O157:H7 and diluted to the final desired CFUs (10^1^–10^8^) in each sample and the assay procedure was kept similar as that of simple buffer.

## Results and Discussion

### Synthesis and Characterization of PNC Nanostructures

Standard synthesis of PNC, as shown in Scheme 1, is composed of two steps: first, the synthesis of poly­(acrylic acid) (PAA) polymer-coated cerium oxide nanoparticles/nanoceria (NC),^43^ followed by *in situ* formulation of GNPs within the PAA coatings of NC, as described in our previously optimized protocol.^44^

Water-based NC formulation is a stepwise method in which the PAA solution is added 30 s after the formation of a CeO_2_ nanocrystal, whereas the core size of CeO_2_ nanocrystals can be tuned by altering the time of PAA solution addition. Similarly, the coating thickness of the polymer is optimized by adding different amounts of PAA during the reaction, keeping the core diameter constant as demonstrated in our previous studies. The physicochemical and morphological characteristics of the CeO_2_ nanoparticle were determined using a combination of different analytical techniques. Figure 2A,B shows the DLS analysis (*D* = 43.1 ± 3 nm and ζ-potential = −31.8 ± 2 mV) of the synthesized NC. The negative zeta potential indicated for PAA coatings was further confirmed by FT-IR spectroscopy with an observed carboxyl stretching band at 1695 cm^–1^ (Figure 2E). The X-ray diffraction (XRD) pattern of NC shown in Figure 2C further indicated nano crystallinity and characteristic Bragg peaks (111), (220), and (311) typical of fluorite structures. High-resolution XPS spectra of NC indicated the presence of Ce 3d core levels and coexistence of Ce^3+^ and Ce^4+^ oxidation states (Figure 2D and Table S1).

**Figure 2:**
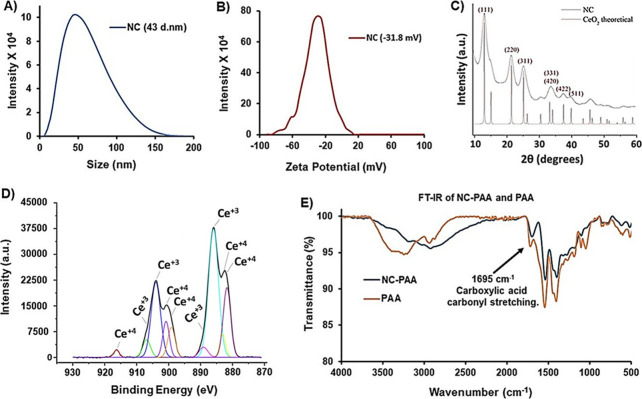
Characterization of nanoceria (NC). (A) Hydrodynamic diameter and (B) ζ-potential of NC. (C) XRD pattern of NC; positions of Bragg peaks confirmed the fluorite structure. (D) High-resolution Ce 3d XPS spectra of CeO_2_. (E) FT-IR spectra of nanoceria (NC-PAA) and PAA polymer for the confirmation of PAA coating on the surface of NC.

Following synthesis of PAA-coated NC, in situ formulation and simultaneous GNP loadings within the PAA coating of NC were achieved in a one-step process by boiling a mixture of PAA-coated CeO_2_ NP and gold chloride (HAuCl_4_) solution in the presence of sodium citrate, as described previously.^44^ In this study, different PNC preparations with 10, 40, and 60% of loaded Au were made by varying the molar ratio of HAuCl_4_ relative to PAA-coated NC while keeping other reaction parameters constant. The resulting Au concentrations within the PAA coatings of NC were analyzed using EDS and have been expressed as a weight percentage. The molar ratio was not increased beyond 60% loaded Au in PNC as those formulations were observed to be unstable and aggregated.

Representative dynamic light scattering (DLS) studies of PNC with 60% gold showed the formation of nanoparticles with an average diameter of 50 ± 3 nm and zeta potential of 34 ± 2 mV (Figure 3A,B). The formulation was found to be wine red in color, monodispersed, and stable with no signs of aggregation. The λ_max_ at 544 nm (LSPR, Figure 3C) attributed to plasmonic absorption of gold nanoparticles further confirmed their successful encapsulation. Surface plasmon resonances (SPR) were found to be similar (λ_max_ = 543–545 nm) for preparations containing varying amounts of Au (10, 40, and 60%), indicating the *in situ* formation of similar sizes of GNPs in these preparations. Figure 3D shows a representative transmission electron microscopy (TEM) image of a PNC solution and energy-dispersive X-ray spectroscopy (EDS).

**Figure 3:**
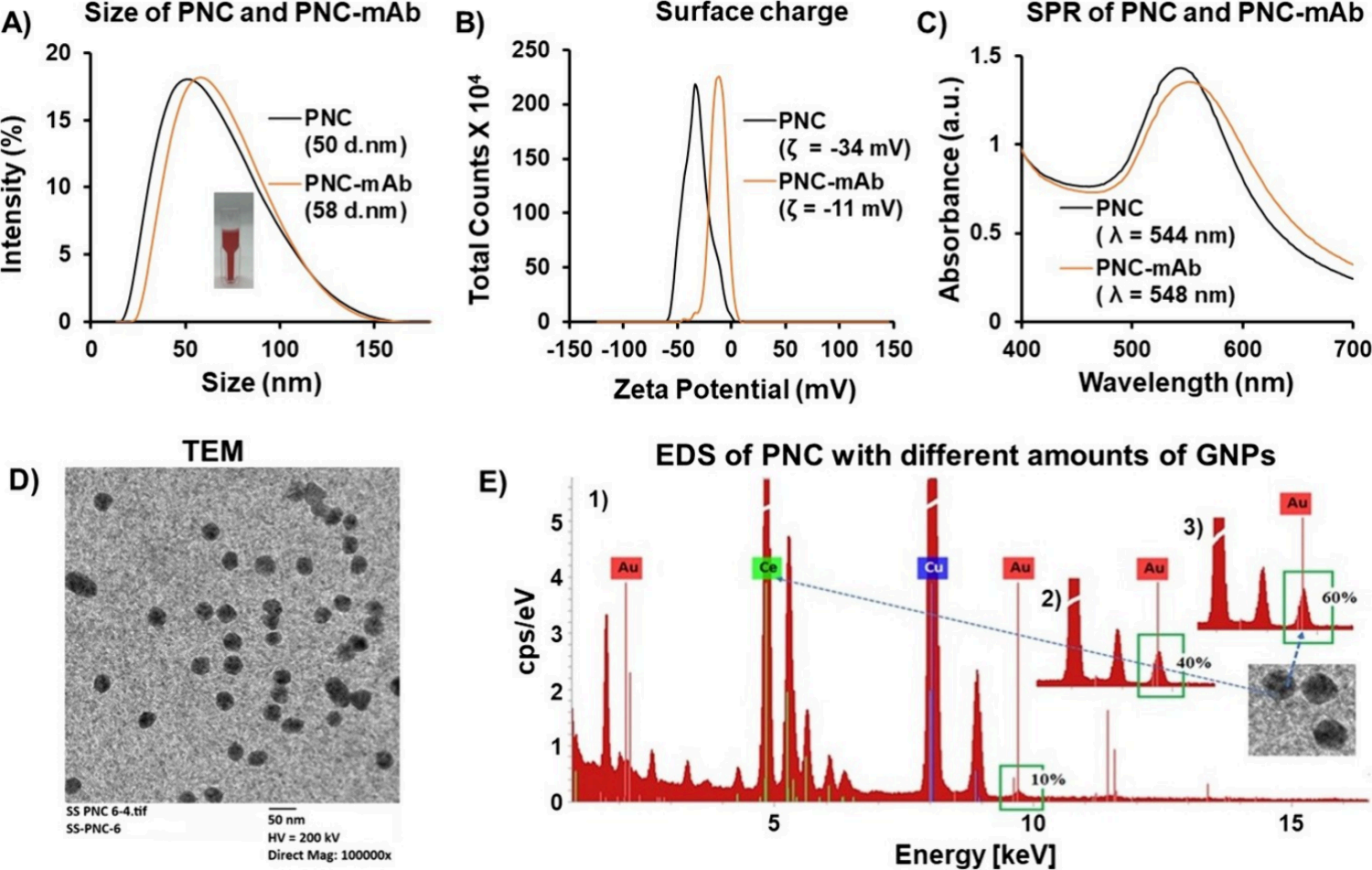
Characterization of plasmonic nanoceria (PNC) and antibody conjugated PNC (PNC–mAb). (A) Hydrodynamic diameter of PNC before and after conjugation. (B) ζ-potential and (C) UV–vis absorption spectra of PNC and PNC–mAb showing successful conjugation. (D) TEM of PNC (scale bar: 50 nm) and (E) EDS of PNC with 10, 40, and 60% encapsulated GNPs.

XRD patterns (Figure 4A) of PNC showed the presence of (111), (220), (311), and (331) planes characteristic of NC and (111) and (220) planes characteristic of metallic Au, confirming the nanocrystallinity and presence of a cubic fluorite structure.^43^ X-ray photoelectron spectroscopy (XPS) experiments were performed to determine the surface composition and chemical speciation of Au and Ce in PNC. XPS spectra (Figure 4B,C) showed the presence of Au 4f and Ce 3d core-level electrons. Comparison of the high-resolution scans of the Ce 3d peak of PNC and NC deconvoluted using a Gauss–Lorentz fitting confirmed the coexistence of Ce^3+^ and Ce^4+^ oxidation states (Table S2) within the samples. The estimated ratios of Ce^3+^/Ce^4+^ were 1.907:1 for PNC and 1.594:1 for NC, as determined from the relative peak areas of the chemical species (Ce^3+^/ Ce^4+^). An increase in surface ratio of Ce^3+^/Ce^4+^ in PNC can be attributed to induced electron transfer from Au to Ce atoms that triggers the conversion of Ce^4+^ to Ce^3+^.^45^ Furthermore, fluorescence emissions of NC and PNC (60% Au) were compared (Figure S1). Weak emissions at 525 nm corresponding to 5d–4f of Ce^3+^ were observed in NC. The presence of plasmonic GNPs resulted in an increase in fluorescence intensity emission in PNC (60% Au). This is attributed to the unique phenomena of optical coupling of fluorescence emission of NC and surface plasmon resonance of embedded GNPs. Next, the synthesized PNCs were functionalized with targeted anti-*E. coli* O157:H7 mAb using the passive conjugation protocol as shown in Scheme 1. The concentration of the antibodies for the synthesis of the PNC–antibody conjugates was optimized as detailed in the Supporting Information and Figure S4. A shift in the size distribution of PNC from 50 to 58 d.nm was observed when conjugated with mAb. The zeta potential for PNC–mAb was found to be ζ = −11 mV versus ζ = −34 mV for PNC, and the SPR results showed upon conjugating mAb a 4 nm band shift confirming the successful conjugation of PNC to the mAb.

**Figure 4:**
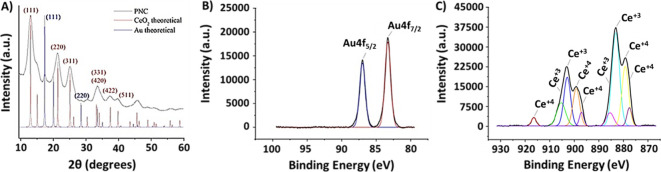
Characterization of PNC. (A) XRD pattern of PNC and (B, C) high-resolution XPS spectra showing the presence of Au 4f and Ce 3d core levels in PNC.

### Peroxidase-Mimetic Activities and Stability Assessment of PNC Nanostructures

The intrinsic peroxidase-like activity of PNC preparations containing 10, 40, and 60% Au was investigated by monitoring the oxidation of 3,3′,5,5′-tetramethylbenzidine (TMB) (λ_max_ = 652 nm, molar extinction coefficient = 39,000 M^–1^ cm^–1^) in the presence of cosubstrate H_2_O_2_ to a blue-colored product. For quantification of catalytic activity, steady-state kinetic parameters of PNC preparations were determined under optimized reaction conditions (pH ∼4.0, 37 °C) as a function of [TMB] substrate (*S*) (Figure 5). The catalytic activity of NC and horseradish peroxidase (HRP) was used for comparison (Table S3). Michaelis–Menten curves were obtained by plotting the initial reaction velocities versus TMB concentrations, and kinetic parameters (*V*_max_ and *K*_m_), catalytic constant (*K*_cat_ = *V*_max_/[*E*]), and catalytic efficiency *K*_cat_/*K*_m_ were determined (Table 1). When *K*_cat_ is compared, data suggest that PNC preparations exhibited 10^3^-fold higher catalytic activity than HRP. Additionally, TMB exhibited excellent affinity toward PNC nanostructures as *K*_m_ values for TMB were found to be smaller than for natural enzyme HRP (Table 1 and Table S3). As shown in Table 1, encapsulated GNPs played a critical role in determining catalytic activity in PNC nanostructures. This is due to the transfer of electrons from the Au atom to ceria’s surface that converts Ce to its reduced state (Ce^3+^), thereby enhancing its catalytic activity. It is worth emphasizing that the highest catalytic activity was observed in PNC preparations containing 60% Au. Hence, it was selected for sensing applications. Also, PNC exhibited ∼100-fold higher catalytic activity than NC (Figure S2 and Table S3). Notably, the *K*_cat_ value of PNC is 5.0 × 10^6^ and similar to top-performing nanozymes, including nickel–platinum and platinum-based enzyme mimics known to exhibit record-high catalytic activities (Table S4). Control experiments using pure GNPs and a physical mixture of GNPs + NC showed that the ultrahigh activity arises exclusively from interfacial synergy (Figure S2) and is not contributed from the individual components. Stability tests conducted on PNC preparations suggested superior pH and thermal stabilities as evidenced by their relative peroxidase-mimetic activities over a range of pH values and temperatures (Figure S3). Catalytic activities were observed to be maximum between pH 4.0–5.0 and at temperatures between 25 and 40 °C.

**Figure 5:**
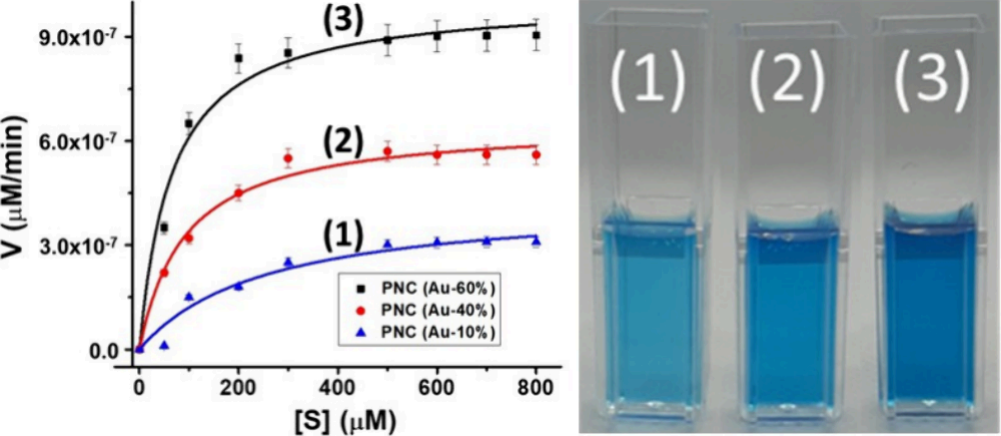
Steady-state kinetics assay of various PNC formulations ((1) PNC Au-10%, (2) PNC Au-40%, and (3) PNC Au-60%). Image showing the peroxidase-like activity of PNCs for TMB oxidation. Error bars represent the standard deviations from five independent measurements.

**Table 1:** Comparison of Catalytic Activities of Various PNC Formulations (PNC Au-10%, PNC Au-40%, and PNC Au-60%) Derived from the Michaelis–Menten Equation

catalyst	*K*_m_ (μM)	*V*_max_ (10^–7^ M/s)	*k*_cat_ (s^–1^)	*k*_cat_/*K*_m_ (M^–1^ s^–1^)
PNC (Au-10%)	256	4.3	2.15 × 10^6^	8.3 × 10^9^
PNC (Au-40%)	92	6.5	3.25 × 10^6^	3.5 × 10^10^
PNC (Au-60%)	66	10	5.0 × 10^6^	7.5 × 10^10^

### Catalytic Mechanism Determination of PNC through Density Functional Theory Calculations

To explore the mechanisms for peroxidase-mimetic activity of PNC and specifically the effect of Au clusters on CeO_2_ nanoparticles (NC), first-principal calculations were conducted on two model structures. One model structure is a series of equidistantly spaced CeO_2_ (111) slabs, each with three layers of Ce atoms and six layers of O atoms, which are separated by 15.7 Å from one another. The other model is the same slab structure with seven extra Au atoms (Au/CeO_2_, PNC) residing on the top surfaces of each slab. The model structures were optimized for the atomic positions of the two layers of Ce and four layers of O atoms on top of each slab, while the bottom Ce and O atoms were fixed. Au atomic positions were also optimized in the Au/CeO_2_ model structure. The optimized model structures are shown in Figure 6. After optimization, Bader’s charge analysis was performed.^46–48^ The calculated Bader’s charges are listed in Table 2. As expected, O is anionic, and Ce is cationic in CeO_2_. When Au atoms are deposited onto the CeO_2_ (111) face, the average Ce Bader’s charge lowers from pure CeO_2_’s + 2.3985 to Au/CeO_2_’s + 2.3749. Meanwhile, Au atoms also bear positive charges. This indicates that Au atoms donate electrons to Ce. We checked all Ce atoms and found, in the immediate vicinity of Au atoms (Figure 6), one specific Ce atom, Ce*, whose charge is much less positive than the average Ce charge (Table 2). This confirms that Au atoms transfer their electrons to some of the Ce atoms close by. We also calculated the density of states (DOS) of Ce atoms in the model structures (Figure 7). The DOS curves of CeO_2_ show that Ce 4f states are largely localized and nearly unoccupied by electrons, as evidenced by the narrow peaks above the Fermi level (*E*_F_). In Au/CeO_2_, for all Ce atoms other than Ce*, the narrow 4f peaks are also above *E*_F_. However, for Ce*, its 4f peaks coincide with *E*_F_. This means that its 4f states are partially occupied by electrons. As learned from Bader’s charge analysis above, the electrons in Ce*’s 4f states are donated by Au atoms. Combining Bader’s charge and Ce DOS analysis, we conclude that there is charge transfer from Au to Ce atoms in CeO_2_, especially the Ce atoms adjacent to Au atoms, such as Ce*. The donated electrons partially populate Ce atoms’ 4f states, which make these Ce atoms more active in catalysis than the other Ce atoms whose 4f states are empty. Overall, these studies indicate that induced electron transfer from the Au to the Ce atom is a critical factor for enhancing PNC’s catalytic activity. A similar kind of metal–support interfacial electronic interactions has been reported recently for copper-based single-atom material that exhibited higher efficacy as an antimicrobial agent due to the presence of atomically dispersed Cu sites.^49^

**Figure 6:**
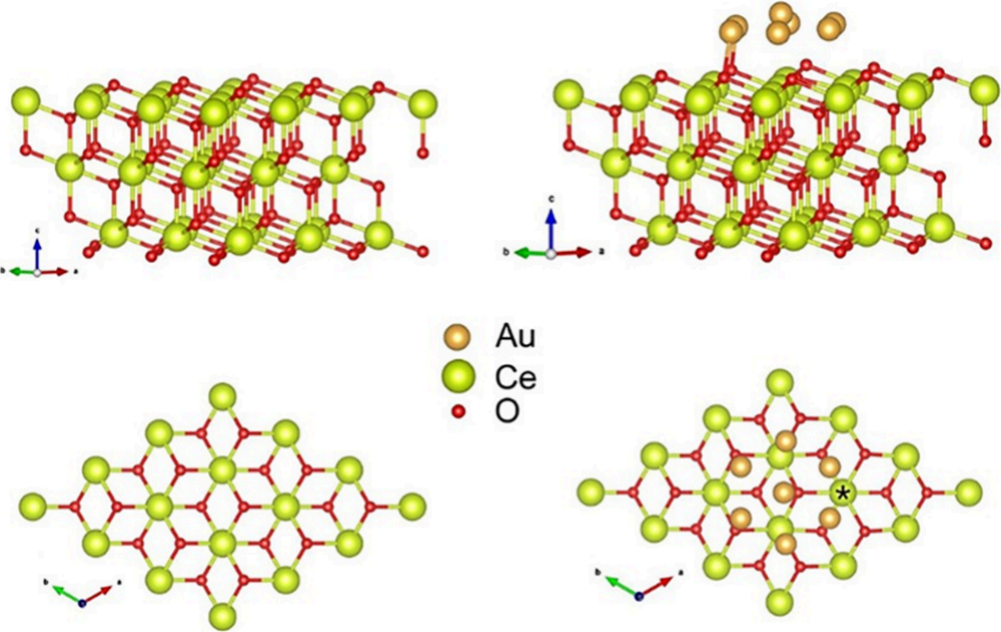
Optimized model structures of CeO_2_ and Au/CeO_2_ (PNC).

**Table 2:** Calculated Average Bader’s Charge^a^

	average Bader’s charge	
atom	CeO_2_ slab	Au/CeO_2_ slab
Ce*	+2.3985	+2.3749
**Ce**		+2.0838
**O**	–1.1992	–1.1914
**Au**		+0.0304

a The “*” indicates the Ce* atom, which received the most significant charge transfer from Au.

**Figure 7:**
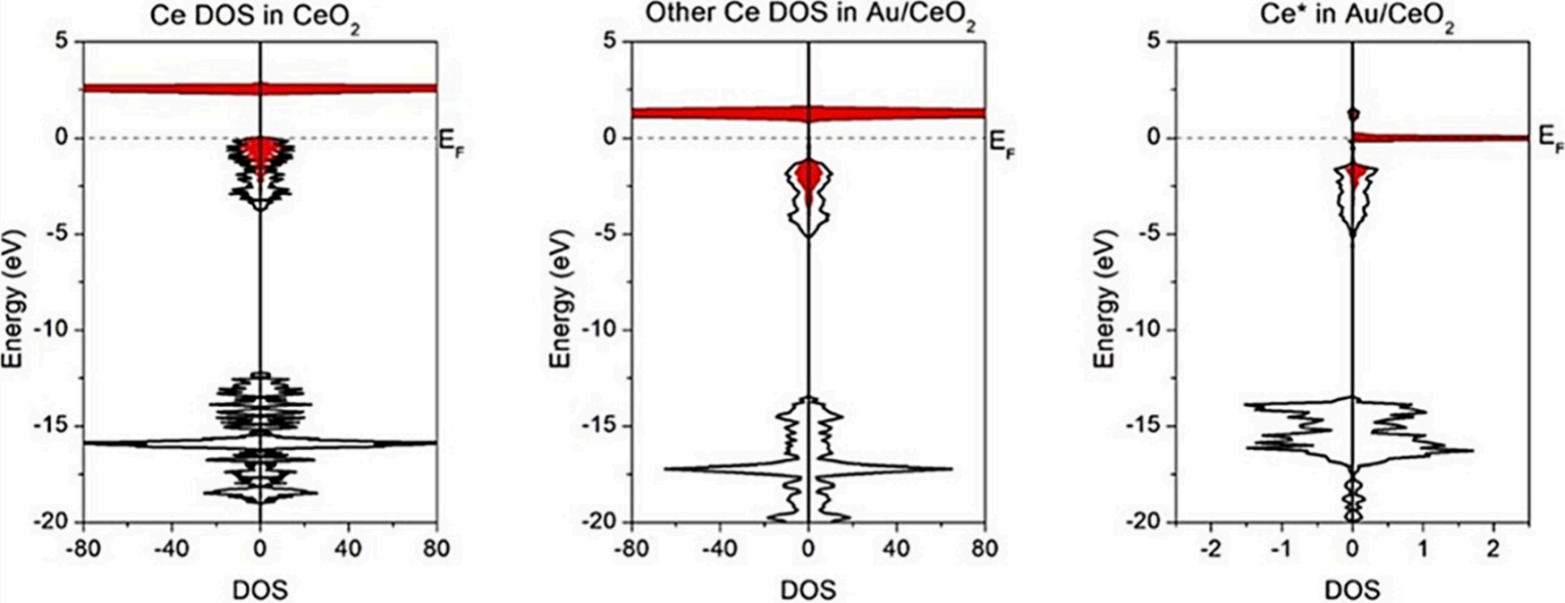
Density of states (DOS) of Ce atoms. The red shaded areas indicate the contribution from Ce 4f states.

### Evaluation of PNC Sensing Performance for *E. coli* O157:H7

Finally, PNC (Au-60%) exhibiting maximum catalytic activity was chosen for the detection of the target pathogen *E. coli* O157:H7. The performance of PNC-based sandwich ELISA was compared to NC and conventional HRP ELISA using the same set of antibodies and similar procedures. Different concentrations (CFU) of *E. coli* O157:H7 were added in a 96-well plate, and absorbance of the blue-colored product at 652 nm, indicating the formation of oxidized TMB, was used as an end point (Figure 8). PNC-based ELISA exhibited a dynamic detection range spanning from 10^1^ to 10^11^ CFU/mL. On the basis of the standard deviation of the residuals and slope of the calibration curve, the limit of detection of PNC-based sandwich ELISA for *E. coli* O157:H7 was 10 CFU/mL.

**Figure 8:**
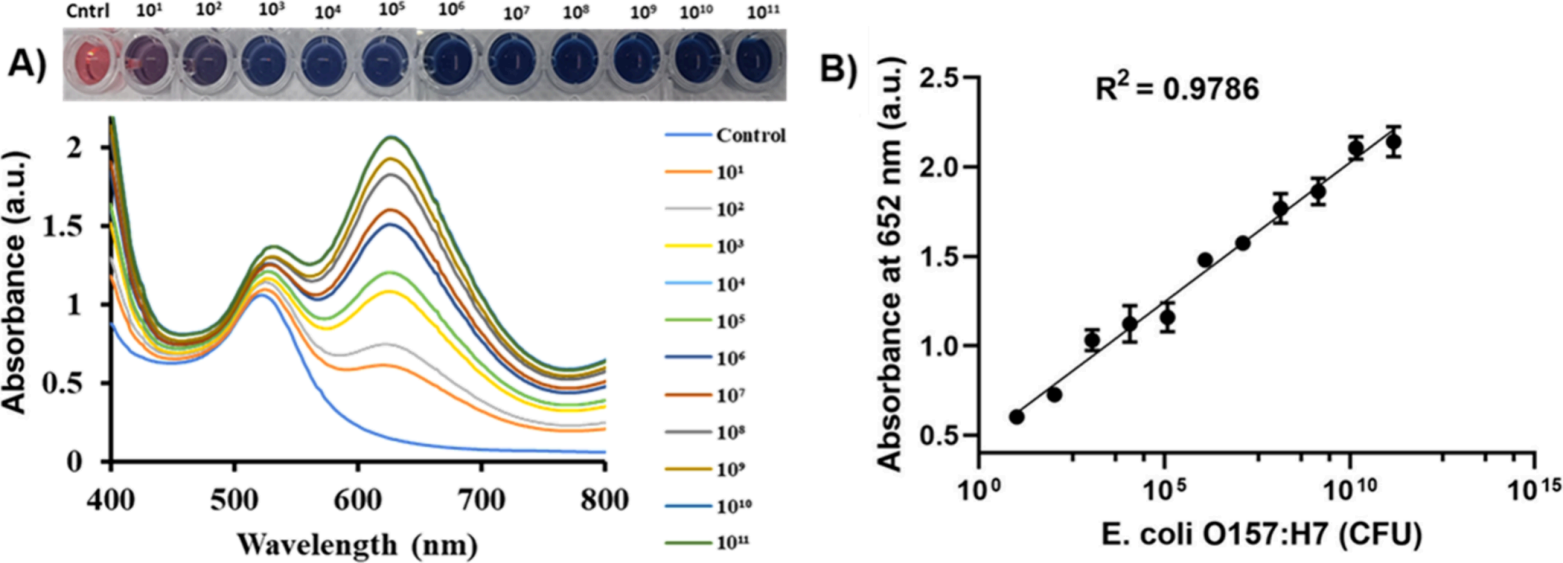
Detection of *E. coli* O157:H7 using (A) PNC-based sandwich ELISA. (B) Calibration curve of the detection results. Error bars represent the standard deviation from five independent measurements.

However, the detection sensitivities of NC and HRP were found to be much lower (Figure S5). The enhanced sensing performance of PNC-based ELISA can be ascribed to their ultrahigh peroxidase-mimetic activity. In addition, the application of PNC-based sandwich ELISA for the detection of *E. coli* O157:H7 in real-world complex food samples such as milk was conducted (Figure S6). Even though the analytical sensitivity was slightly reduced due to the presence of high fat and other protein contaminants, it was not substantially affected in the milk sample. The bacterial CFUs that were accurately detected in concentrations were as low as 10^3^ CFU/mL.

## Conclusions

In summary, the present work introduces an innovative approach for accelerating the redox cyclability in metal-based nanozymes for overcoming their limited catalytic efficiency and provides a new avenue of achieving robust enzyme-free signal amplification. Synthesized redox-active PNC nanostructures exhibited ultrahigh catalytic activity, which was attributed to accelerated redox cycling between Ce^3+^ and Ce^4+^ induced via electron transfer from Au to the Ce atom. PNC-based peroxidase mimics, when applied for the ELISA testing of *E. coli* O157:H7, achieved substantially higher detection sensitivity than the conventional assay employing natural enzymes.

## Supplementary Material



## Notes

Fluorescence emission intensity spectra of NC and PNC, kinetic plots of NC and HRP, plots of sandwich ELISA of NC and HRP, stability tests at different pH and temperatures, flocculation curves of antibody immobilization on the surface of PNC, XPS data table of NC and PNC containing details on the peak position and peak height, and comparison of kinetic parameters and catalytic efficiencies of NC and HRP (PDF)
