# Supplementary material for: Interfacial Redox Recycling Nanocatalysts with Ultrahigh Peroxidase Activity for Colorimetric Sensing Applications
Source: ACS Appl Nano Mater. 2026 May 29;9(23):10887–95. doi: 10.1021/acsanm.6c01249 (PMC13270462; doi:10.1021/acsanm.6c01249)
Supplement: Supplementary file 1 [file an6c01249_si_001.pdf]

“Supporting Information”

Interfacial Redox Recycling Nanocatalysts with Ultrahigh Peroxidase Activity for Colorimetric Sensing Applications

Santimukul Santra,[1,2] Eniola Arogunyo,[2] Rahab Kanogo,[1] Abigail Teitelbaum,[1] Caroline Gichuru,[1] Fei Wang,[1] Rishi Patel,[3] and Tuhina Banerjee[1]**

[1]Department of Chemistry and Biochemistry, Missouri State University, 901 S. National Avenue, Springfield, MO 65897, United States of America

[2]Department of Chemistry, Pittsburg State University, 1701 S. Broadway Street, Pittsburg, KS 66762, United States of America

[3]Jordan Valley Innovation Center, Missouri State University, 542 N. Boonville Avenue, Springfield, MO 65806, United States of America

*Corresponding authors Tuhina Banerjee, Email: tbanerjee@missouristate.edu and Santimukul Santra, Email: ssantra@missouristate.edu

Peak Index	Peak Position (eV)	FWHM	Peak Height	Peak Area
Ce^{3+}	885.58	32475.40	3.34	115624.42
Ce^{3+}	887.73	4591.35	3.20	15660.75
Ce^{3+}	903.89	16973.02	2.97	53746.76
Ce^{3+}	906.18	8091.19	3.90	33602.60
Ce^{4+}	880.43	6348.84	2.18	14728.13
Ce^{4+}	882.00	20822.20	2.92	64819.21
Ce^{4+}	898.49	4766.95	1.94	9827.55
Ce^{4+}	900.48	12996.09	2.99	41311.63
Ce^{4+}	916.51	2892.35	2.11	6502.45

Table S1: Peak position, peak heights, and peak area of Ce^{3+} and Ce^{4+} species in NC.

Peak Index	Peak Position (eV)	FWHM	Peak Height	Peak Area
Ce^{3+}	885.28	3.72	37885.89	150126.05
Ce^{3+}	888.47	3.21	2972.05	10163.23
Ce^{3+}	903.68	3.48	22175.75	82037.93
Ce^{3+}	906.62	2.91	5180.96	16032.40
Ce^{4+}	880.98	2.84	20578.93	62287.05
Ce^{4+}	882.34	2.12	6838.64	15466.02
Ce^{4+}	898.69	2.71	8849.28	25510.45
Ce^{4+}	900.42	2.43	10609.46	27405.88
Ce^{4+}	916.24	2.08	2166.17	4791.36
Au $4f_{7/2}$	83.31	1.00	17883.69	19108.62
Au $4f_{5/2}$	86.99	0.99	13481.41	14185.48

Table S2: Peak position, peak heights, and peak area of Ce^{3+} and Ce^{4+} and Au^0 species in PNC containing 60% Au.

Catalyst	K_m (μM)	V_{max} (10^{-8} M/s)	k_{cat} (s^{-1})
**GNP**	536	4.3	0.1 X 10^4
**NC**	467	5.8	0.6 X 10^4
**NC + GNP**	321	2.1	0.1 X 10^4
**HRP**	366	3.7	1.3 X 10^3

Table S3: Catalytic activities of NC, HRP, GNP and NC + GNP derived from Michaelis Menten equation.

Comparison of kinetic parameters of other peroxidase-mimetic nanozymes towards substrate TMB.

Nanozyme	Km(mM)	V_{max} (Ms^{-1})	K_{cat} (s^{-1})	Ref.
**a)** HRP	0.434	10.0 x 10^{-8}	4 x 10^{3}	[S1]
**b)** Fe_3O_4	0.098	3.4 x 10^{-8}	3 x 10^{4}	[S1]
**c)** Prussian blue nanoparticles	0.6	~ 10^{-7}	10^{3}-10^{4}	[S2]
**d)** Ni-Pt	35.0	2.6 x 10^{-5}	4.5 x 10^{7}	[S3]
**e)** Porous NC	0.147	6.2 x 10^{-5}	5.3 x10^{4}	[S4]
**f)** AuPt NPs	0.42	73.2 x 10^{-8}	4.2 x 10^{6}	[S5]
**g)** PNC	0.092	6.5 x 10^{-7}	3.2 x10^{6}	[This work]

Table S4: Comparison of kinetic parameters of other peroxidase-mimetic nanozymes towards substrate TMB.

Detection Technique	Limit of detection	Response time	Ref.
CdTe/CdS Quantum Dot-Labeled Fluorescent LFA	10^{4} CFU mL^{-1}	12 min	[S6]
CRISPR-Cas9 based Isothermal Amplification Method	4.0 × 10^{1} CFU mL^{-1}	under 60 min	[S7]
RT-PCR kit	2.6 × 10^{4} mL^{-1}	~ 24 h	[S8]
Electrochemical impedance	6.0 × 10^{3} mL^{-1}	~ 30 min	[S9]
Microfluidic droplet digital PCR	10 CFU mL^{-1}	~ 2 h	[S10]
Our method	10 CFU mL^{-1}	~ 15 min	

Table S5: Comparison of the detection performance of PNC-based ELISA with other sensing methods.

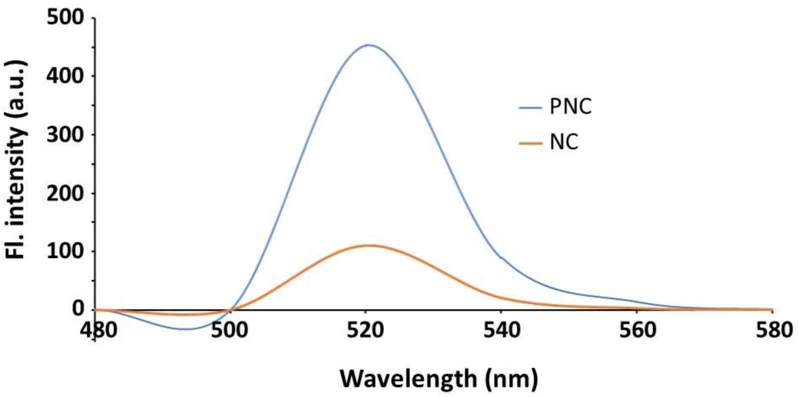

Figure S1: Fluorescence emission spectra of NC and PNC.

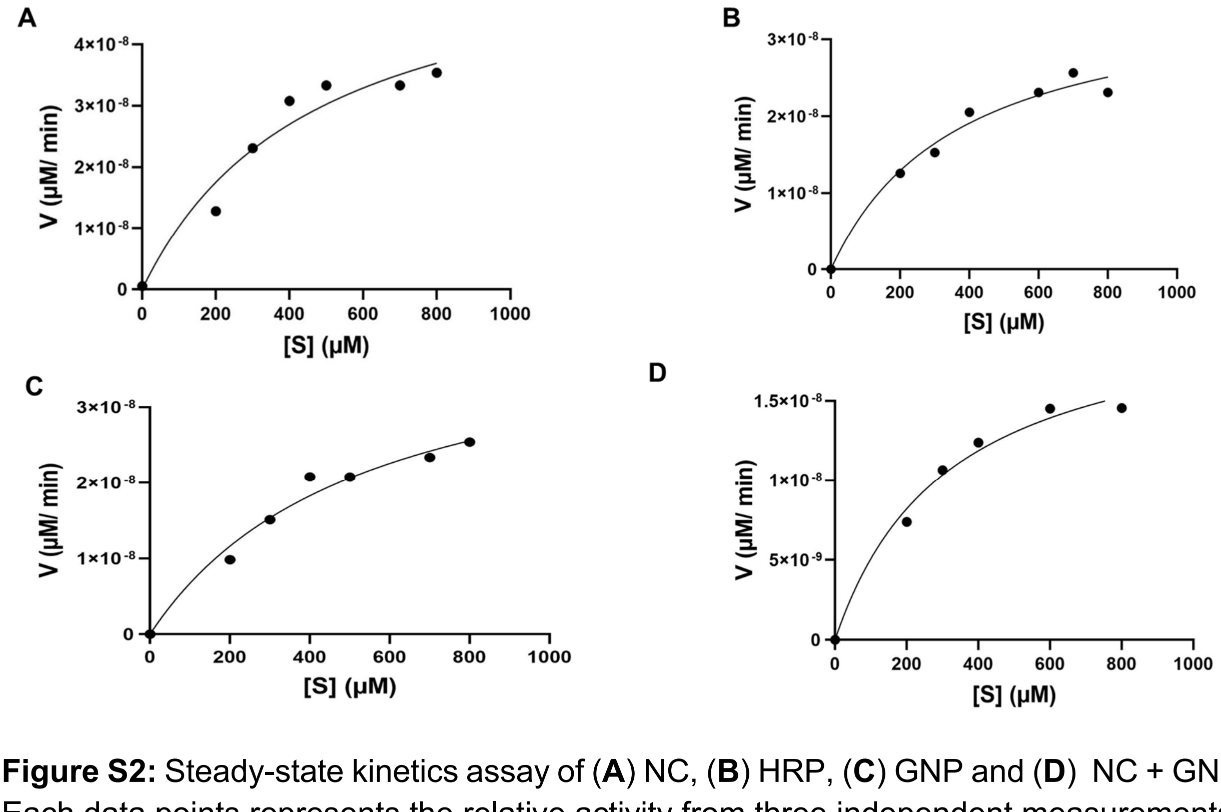

Figure S2: Steady-state kinetics assay of (**A**) NC, (**B**) HRP, (**C**) GNP and (**D**) NC + GNP. Each data points represents the relative activity from three independent measurements.

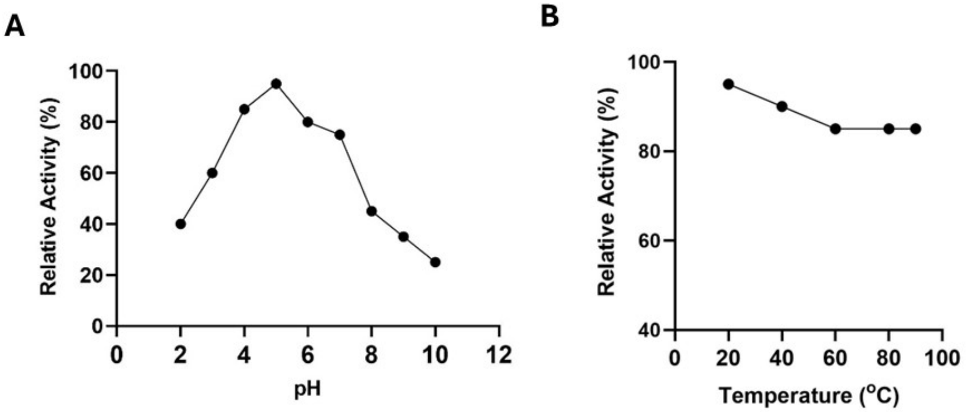

Figure S3: Stability tests of PNC over a range of **(A)** pH values and **(B)** temperatures**.** Each data points represents the relative activity from three independent measurements

Flocculation Curve: Optimization of detection antibody concentration for optimizing stability of PNC-antibody conjugates

Stabilizing concentration of antibodies for conjugation was determined through flocculation experiments. As shown in **Figure S4**, conjugation of different concentrations of mAbs to PNC did not result in aggregation. However, after addition of 10% NaCl, a concentration-dependent change in OD_{580} was observed, indicating flocculation of PNC. Flocculation was observed between 0-4 µg/mL of mAb, and in comparison, PNC conjugates synthesized with 16 µg/mL of mAb were stable and red in color. Conjugates that turned blue after the addition of 10% NaCl due to aggregation were considered unstable and not selected for ELISA experiments. The absorption maximum of PNC conjugate (16 µg/mL) shifted about 4 nm, which is attributed to the change in local refractive index on the surface of PNC after conjugation. The appearance of a visible line on the protein A/G test strips was further used for the confirmation of conjugation.

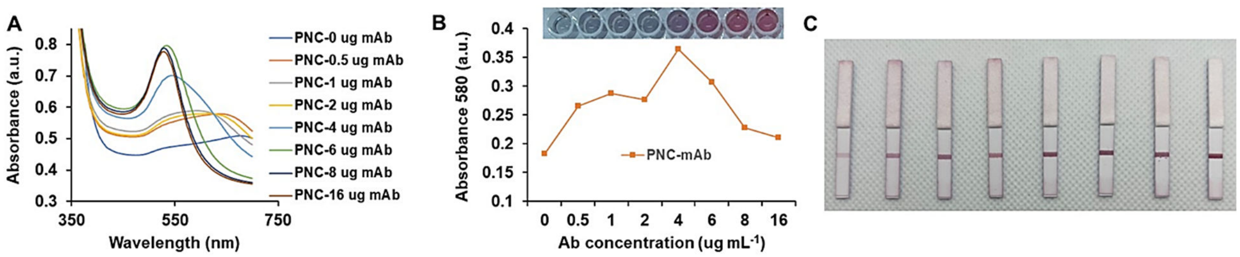

Figure S4: (A) UV-Vis trace of PNC-mAb conjugates at different antibody concentrations. **(B)** Flocculation curve of PNC-mAb conjugates at different antibody concentrations, inset corresponding images after addition of 10% NaCl. **(C)** A/G Strip Test for the confirmation of conjugation.

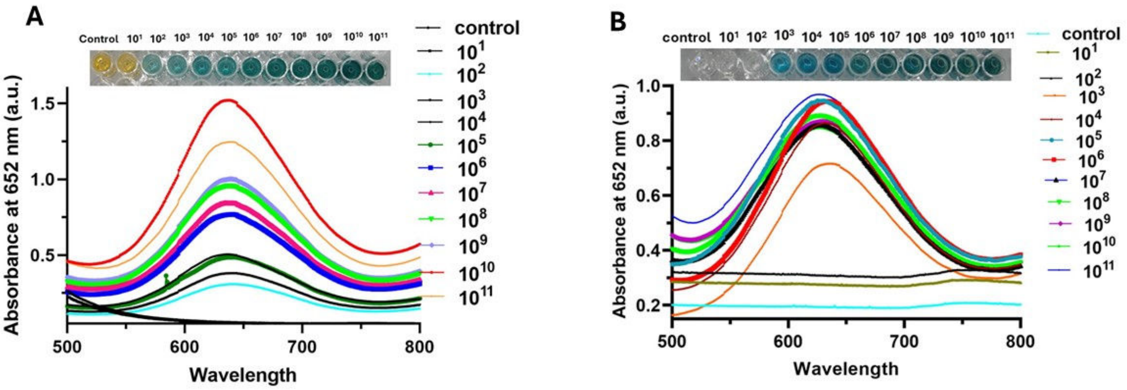

Figure S5: Detection of E. coli O157:H7 using (**A**) NC and (**B**) HRP-based sandwich ELISA.

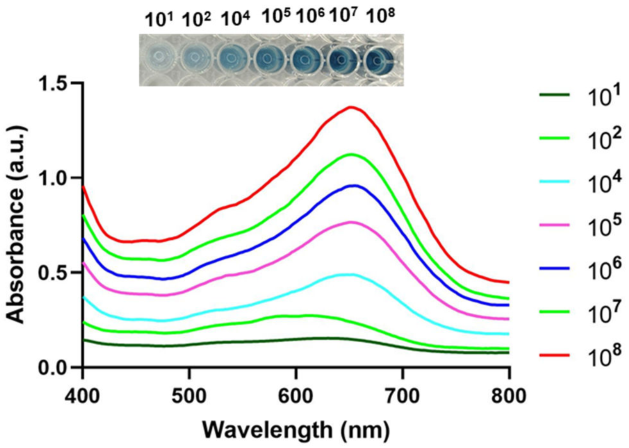

Figure S6: Detection of E. coli O157:H7 in milk using PNC-based sandwich ELISA.

References:

[S1] Gao, L.; Zhuang, J.; Nie, L.; Zhang, J.; Zhang.; Y, Gu N. et al. Intrinsic peroxidase-like activity of ferromagnetic nanoparticles. *Nat. Nanotechnol*. **2007**, *2,* 577–583.

[S2] Komkova, MA.; Karyakina, EE.; Karyakin, AA. Catalytically Synthesized Prussian Blue Nanoparticles Defeating Natural Enzyme Peroxidase. *J. Am. Chem. Soc.* **2018**, *12,* 11302-11307.

[S3] Xi, Z.; Wei, K.; Wang, Q.; Kim, M. J.; Sun, S.; Fung, V.; Xia, X. Nickel-Platinum Nanoparticles as Peroxidase Mimics with a Record High Catalytic Efficiency. *J. Am. Chem. Soc.* **2021**, *143,* 2660.

[S4] Tian, Z. M.; Li, J.; Zhang, Z. Y.; Gao, W. M.; Zhou, X. Q.; Qu, Y. Q. Highly sensitive and robust peroxidase-like activity of porous nanorods of ceria and their application for breast cancer detection. *Biomaterials* **2015**, *59*, 116–124.

[S5] Nuti, S.; Fernández-Lodeiro, J.; Palomo J. M.; Capelo-Martinez J. L.; Lodeiro, C.; Fernández-Lodeiro, A. Synthesis, Structural Analysis, and Peroxidase-Mimicking Activity of AuPt Branched Nanoparticles. *Nanomaterials* **2024,** *14*,1166.

[S6] Yu J., Su J., Zhang J., Wei X., Guo A. CdTe/CdS Quantum Dot-Labeled Fluorescent Immunochromatography Test Strips for Rapid Detection of Escherichia coli O157:H7. *RSC Adv*. **2017**, 7,17819–17823.

[S7] Sun X., Wang Y., Zhang L., Liu S., Zhang M., Wang J., Ning B., Peng Y., He J., Hu Y., et al. CRISPR-Cas9 Triggered Two-Step Isothermal Amplification Method for E. coli O157:H7 Detection Based on a Metal–Organic Framework Platform. *Anal. Chem*. **2020**, 92, 3032–3041.

[S8] Ibekwe, A. M.; Watt, P. M.; Grieve, C. M.; Sharma, V. K.; Lyons, S. R. Multiplex fluorogenic real-time PCR for detection and quantification of Escherichia coli O157:H7 in dairy wastewater wetlands. *Appl. Environ. Microbiol.* **2002**, *68* , 4853– 62.

[S9] Ruan, C.; Yang, L.; Li, Y. Immunobiosensor chips for detection of Escherichia coli O157:H7 using electrochemical impedance spectroscopy.*Anal. Chem.* **2002**, 74,4814-4820.

[S10] Bian X., Jing F., Li G., Fan X., Jia C., Zhou H., Jin Q., Zhao J. A Microfluidic Droplet Digital PCR for Simultaneous Detection of Pathogenic Escherichia coli O157 and Listeria Monocytogenes. *Biosens. Bioelectron*. **2015**;74:770–777.
